# Concentration Effect of Reducing Agents on Green Synthesis of Gold Nanoparticles: Size, Morphology, and Growth Mechanism

**DOI:** 10.1186/s11671-016-1393-x

**Published:** 2016-04-27

**Authors:** Hyun-seok Kim, Yu Seon Seo, Kyeounghak Kim, Jeong Woo Han, Youmie Park, Seonho Cho

**Affiliations:** National Creative Research Initiatives (NCRI) Center for Isogeometric Optimal Design, Seoul National University, 1 Gwanak-ro, Gwanak-gu, Seoul, 151-744 Republic of Korea; College of Pharmacy, Inje University, 197 Inje-ro, Gimhae, 621-749 Republic of Korea; Department of Chemical Engineering, University of Seoul, Seoul, 130-743 Republic of Korea

**Keywords:** Aggregation mechanism, Concentration of reducing agent, DFT calculation, Gold nanoparticle, Green synthesis, Molecular dynamics

## Abstract

Under various concentration conditions of reducing agents during the green synthesis of gold nanoparticles (AuNPs), we obtain the various geometry (morphology and size) of AuNPs that play a crucial role in their catalytic properties. Through both theoretical and experimental approaches, we studied the relationship between the concentration of reducing agent (caffeic acid) and the geometry of AuNPs. As the concentration of caffeic acid increases, the sizes of AuNPs were decreased due to the adsorption and stabilizing effect of oxidized caffeic acids (OXCAs). Thus, it turns out that optimal concentration exists for the desired geometry of AuNPs. Furthermore, we investigated the growth mechanism for the green synthesis of AuNPs. As the caffeic acid is added and adsorbed on the surface of AuNPs, the aggregation mechanism and surface free energy are changed and consequently resulted in the AuNPs of various geometry.

**Electronic supplementary material:**

The online version of this article (doi:10.1186/s11671-016-1393-x) contains supplementary material, which is available to authorized users.

## Background

Development of new materials with extraordinary and outstanding performance leads to the improvement of engineering devices. Study of the mechanical properties of nanoscale materials has been of significant interest to researchers due to nano-technological development. Nanomaterials such as nanoparticles, nanowires, and nanotubes have a potential to exhibit extraordinary combinations of properties. Due to their remarkable mechanical, electrical, thermal, and magnetic properties, nanomaterials have drawn considerable attention to the scientific community for the past decades.

Nanoparticles have been used in a variety of research fields, including drug delivery, catalysis, bio-sensing, and imaging, due to their numerous potential applications. In naval architecture and ocean engineering, by using nanoparticles as carriers and anchors for antifouling ligands, antifouling can be achieved by slowing the growth of subaquatic organisms [1]. Especially, AuNPs possess numerous advantages, such as low cytotoxicity, facile modification of their surfaces, straightforward synthetic processes, and excellent biocompatibility [2, 3]. The most common method for the synthesis of AuNPs uses chemical reducing agents to convert Au ions to AuNPs. These nanoparticles synthesized by chemical and physical approaches might potentially be harmful to the environment and living organisms [4, 5]. Current sustainability initiatives require the use of green (eco-friendly or environment-friendly)-reducing agents for the synthesis of AuNPs. The green reducing agents include a large number of natural products. Examples of green-reducing agents for the synthesis of AuNPs include plant extracts, phytochemicals, polysaccharides, and microorganisms [6, 7].

The particle size and morphology play crucial roles in controlling the physical, chemical, optical, and electronic properties of these materials [8, 9], and for the synthesis of nanoparticles of designed size and shape, surface chemistry, morphology, and mechanism research were imperative [10]. Intense researches have been made to control the size of nanoparticles and their distribution by varying the size of particle seed [11], the concentration of chemical reducing agent [12], and temperature [13] through experimental approaches. However, a detailed investigation of the structural and the growth mechanism of AuNPs under green-reducing agent has not been fulfilled yet. In the current study, caffeic acid (3,4-dihydroxy cinnamic acid) from plants was used for the green synthesis of AuNPs. The objectives were (1) to evaluate the green synthesis of AuNPs with various concentrations of caffeic acid and (2) to characterize the prepared AuNPs using UV-visible spectrophotometry, high-resolution transmission electron microscopy (HR-TEM), and measurements of the mean particle size and zeta potential. Furthermore, (3) to elucidate the effect of reducing agent concentration on the aggregation of nanoparticles, we performed DFT and MD simulation to investigate the position and distribution of caffeic acid and interaction with AuNPs at various concentrations. Our experimental and computational results provide a fundamental and geometric understanding of AuNPs at different concentrations of caffeic acid. The developed method suggests an efficient way for synthesizing the desired geometry of AuNPs.

This paper includes both experimental and computational approaches. The morphology and size of green-synthesized gold nanoparticles can be controlled via the concentration conditions of a reducing agent. Due to the adsorption and stabilizing effects, the size of gold nanoparticles decreases at an intermediate concentration. This is attributed to its significant influence of the interfacial properties of nanoparticles such as surface energy and adsorption energy.

## Methods

### Experiment Details

#### Chemicals and Instrumentation

Hydrochloroauric acid trihydrate (HAuCl_4_·3H_2_O) and caffeic acid were purchased from Sigma-Aldrich (St. Louis, MO, USA). All other reagents were of analytical grade. Deionized water was used to prepare all of the solutions. The UV-visible spectra were acquired with a Shimadzu UV-2600 spectrophotometer (Shimadzu Corporation, Kyoto, Japan). A JEM-3010 electron microscope was used to obtain the HR-TEM images at 300 kV (JEOL, Tokyo, Japan). The mean particle size by dynamic light scattering and zeta potential were measured on a Brookhaven 90Plus at 20 °C (Brookhaven Instruments Co., Holtsville, NY, USA).

#### Green Synthesis of AuNPs with Various Concentrations of Caffeic Acid

The green synthesis of AuNPs with caffeic acid as a reducing agent was performed under a fixed final concentration (0.2 mM) of hydrochloroauric acid trihydrate. While stirring (340 rpm, 220 °C) on a hotplate for 2 min, 200 *μ*L of hydrochloroauric acid trihydrate (1 mM) was added to 800 *μ*L caffeic acid (variable concentration). The reaction mixture (1 mL) was stirred on a hotplate for an additional 1 min and placed in an oven (85 °C) for 12 h. In total, 11 final concentrations of caffeic acid (0.008, 0.04, 0.08, 0.12, 0.16, 0.2, 0.24, 0.28, 0.4, 0.48, and 0.56 mM) were tested for the green synthesis of AuNPs.

### Computation Details

#### Molecular Dynamics

**Fig. 1:**
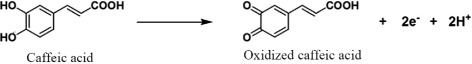
Redox reaction of caffeic acid

In this study, caffeic acid is used as a green-reducing agent to AuNPs. The redox reaction of caffeic acid is illustrated in Fig. 1. Due to the limit of molecular dynamic (MD) simulations, we assume that the caffeic acid is oxidized into oxidized caffeic acids (OXCA) while the resulting electrons (e^−^) are already used to fully neutralize gold ions (Au^3+^) in hydrochloroauric acid trihydrate. Therefore, in our MD simulations, we will only consider the qualitative concentration of OXCAs compared to the experiments with neutralized gold ions to understand the behavior of AuNPs formation by caffeic acids. The OXCAs are modeled from caffeic acid by removing two hydrogens via VMD TopoTools [14].

Bonded and non-bonded interactions of OXCAs are described by the general AMBER force field (GAFF) [15] as shown in Eq. 1

(1) $$\begin{array}{@{}rcl@{}} E_{total} &=& \sum\limits_{bonds} {K_{r} \left({r - r_{0}} \right)^{2}} + \sum\limits_{angles} {K_{\theta} \left({\theta - \theta_{0}} \right)^{2}} \\ &&+ \sum\limits_{dihedrals} {K_{\varphi,n} \left[ {1 + \cos \left({n\varphi - \gamma} \right)} \right]}  \\ && + \sum\limits_{i < j} {4\varepsilon_{i,j} \left[ {\left({\frac{{\sigma_{ij} }}{{r_{ij} }}} \right)^{12} - \left({\frac{{\sigma_{ij} }}{{r_{ij} }}} \right)^{6}} \right]} + \sum\limits_{i < j} {\frac{{q_{i} q_{j} }}{{r_{ij} }}}.\qquad \end{array} $$

The bonded interaction parameters *K*_*r*_,*K*_*θ*_, and *K*_*φ*,*n*_ are force constants. *r*_0_ and *θ*_0_ are equilibrium bond length and angle, respectively. Periodicity and phase angle are represented as *n* and *γ*. Note that the summation over dihedral bonds includes both conventional and improper dihedrals. For the non-bonded interactions, the Lennard-Jones (L-J) parameters of *ε* and *σ* are introduced to describe repulsive and attractive contributions. The final term in Eq. 1 stands for the Columbic pairwise interaction where *q* is the charge of atom. To assign initial bonded and non-bonded force field parameters from the GAFF, the open source antechamber [16] is utilized. During this step, atomic point charges were calculated via antechamber using AM1-BCC [17] method. The water molecules were described using the TIP3P model [18] with a long-range Coulombic solver. To describe the interactions between gold-gold, we applied embedded atom method (EAM) potential [19]. For non-bonded interactions between different types of atoms, Lorentz-Berthelot mixing rules are used to calculate *ε*_*ij*_ and *σ*_*ij*_ as

(2) $$\begin{array}{@{}rcl@{}} \varepsilon_{ij} = \sqrt {\varepsilon_{ii} \varepsilon_{jj} }, \end{array} $$

(3) $$\begin{array}{@{}rcl@{}} \sigma_{ij} = \frac{{\sigma_{ii} + \sigma_{jj} }}{2}. \end{array} $$

#### MD Simulations

All MD simulations are performed by the open source code of LAMMPS (large-scale atomic/molecular massively parallel simulator) [20]. To construct an initial low-density system, the open source simulation setup tool of Packmol [21] is used. The electrostatics were treated using the particle-particle particle-mesh (P^3^M) technique [22]. The SHAKE constraints scheme [23] was applied to water molecules to keep the bond length and angle to the specified values. With reference to a prior MD simulation study on caffeic acids [24] which constrained all the covalent bonds for hydrogen atoms using a linear constraint solver, the oxidized caffeic acids are considered as an independent rigid body.

Using the Packmol package, a given concentration of OXCAs, gold atoms, and water molecules are randomly placed into a cube with the size of 60 by 60 by 60 Angstroms. In this study, 12 cases are simulated for the green synthesis of AuNPs by varying the number of OXCAs from 1 to 24. For each case, there are 1, 2, 3, 4, 5, 6, 7, 8, 9, 10, 20, and 24 oxidized caffeic acids with a fixed number of 100 gold atoms and 2000 water molecules. Additional file 1: Figure S1 a–f shows the randomly packed initial configurations of each case, where the carbon atoms in OXCAs are colored in blue.

After packing the molecules randomly, an energy minimization of the corresponding system is conducted with a steepest descent algorithm. Furthermore, to remove instability of the system, an isothermal isobaric (NPT) simulation is conducted for 1 ps at 358.15 K (85 °C) and 1 bar with the timestep of 0.1 fs. Then, subsequent NPT simulations (358.15 K, 1 bar) were started from these initial systems. The terminal NPT simulation time was 0.5 ns with the timestep of 1 fs. For each case, five independent MD simulations are performed with various random initial velocities. The mean diameter of gold clusters is weighted by the number of gold atoms consisting a cluster and measured at the terminal time as shown in Eq. 4, where $d_{i_{1} }$ and $d_{i_{2} }$ are the two largest diameters of a cluster *i*. In this paper, a cluster consisting of even one gold atom is considered and the atomic radius of gold [25] is utilized for measuring the diameter. The measured weighted mean diameter of gold clusters are averaged over five independent MD simulations for each case.

(4) $$ {\fontsize{7.99pt}{6pt}{}{\begin{aligned} d_{weighted} = \sum\limits_{i = 1}^{N_{clusters}} {\frac{{\left({\frac{{d_{i_{1}} + d_{i_{2}} }}{2}} \right) \times N_{number\,of\,gold\,atoms\,consisting\,the\,cluster} }}{{N_{gold\,atoms\,in\,simulation\,box} }}} \end{aligned}}}  $$

#### DFT Calculation

We performed DFT calculations using the Vienna ab initio Simulation Package (VASP) [26, 27]. The exchange-correlation energies were treated by Perdew-Burke-Ernzerhof (PBE) functional based on a generalized gradient approximation (GGA) [28]. The cutoff energy of 400 eV was used for the expansion of wave functions into plane waves with Brillouin zone at the 2×2×1 Monkhorst-Pack [29] k-point mesh. Total energy calculations were implemented using the residual minimization method to determine the electronic ground-state. The electronic density of states were calculated by the Methfessel-Paxton method with the smearing width of 0.1 eV [30]. Geometries relaxations are conducted by the conjugate gradient algorithm until the all the unconstrained atomic forces were less than 0.03 eV/A. For the calculations of energetics on Au surfaces, we used 5-layer slabs with (3×3) surface unit cells for (111), (110), and (100), respectively (Additional file 1: Figure S3). Due to the large size of OXCA molecules, for the calculations of OXCA adsorption, we doubled the surface unit cells with a slab thickness of 3-layers and 1×1×1 k-point. The vacuum thickness of 15 A was used. To consider van der Walls interactions, we employed DFT-D3 method developed by Grimme [31]. The total energy of an isolated OXCA molecule was calculated in a 20×20×20A simulation box.

## Results and Discussion

### Color of AuNP Solutions and UV-Visible Spectra

**Fig. 2:**
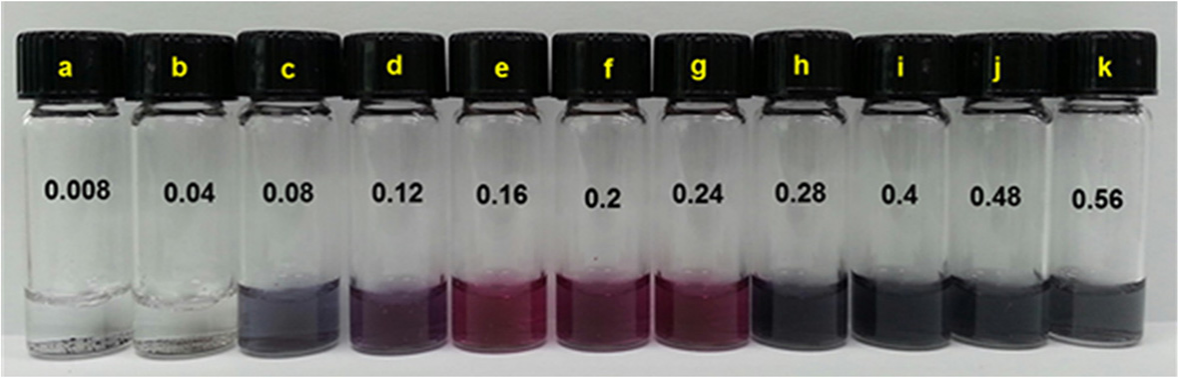
Digital images of AuNPs. The final concentration of caffeic acid is **a** 0.008, **b** 0.04, **c** 0.08, **d** 0.12, **e** 0.16, **f** 0.2, **g** 0.24, **h** 0.28, **i** 0.4, **j** 0.48, and **k** 0.56 mM. The final concentration of Au^3+^ ions is fixed at 0.2 mM. The detailed reaction procedure is described in the experimental section

**Fig. 3:**
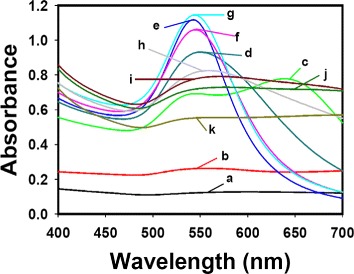
UV-visible spectra of AuNPs. The final concentrations of caffeic acid are **a** 0.008, **b** 0.04, **c** 0.08, **d** 0.12, **e** 0.16, **f** 0.2, **g** 0.24, **h** 0.28, **i** 0.4, **j** 0.48, and **k** 0.56 mM

The formation of AuNPs is easily detected by a change in the color of the solution. In our experiments, no visible change in color was detected for caffeic acid solutions at the lowest tested concentrations of 0.008 and 0.04 mM (Fig. 2a, b). However, at higher concentrations, the color of the solution changed to pale gray, violet, wine, purple, or gray (Fig. 2–k). The UV-visible spectrum obtained for the 0.008 mM solution of caffeic acid did not demonstrate any significant absorbance (Fig. 3a). A slight absorbance was detected in the range of 500–580 nm with 0.04 mM caffeic acid, as shown in Fig. 3b. The characteristic surface plasmon resonance (SPR) band for AuNPs was clearly observed in the range of 500–580 nm in the UV-visible spectra for caffeic acid solutions of 0.08–0.56 mM (Fig. 3c–k).

### Mean Particle Size and Zeta Potential Measurements

**Fig. 4:**
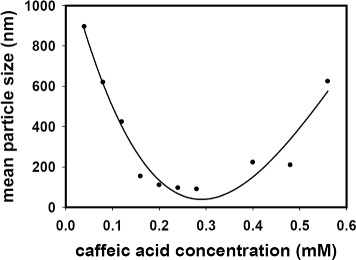
The relationship between mean particle size (nm) and caffeic acid concentration (mM)

The mean particle size (hydrodynamic size) was measured based on dynamic light scattering, and the results are shown in Table 1 and Fig. 4. Over the range of tested concentrations of caffeic acid (0.008 ∼0.56 mM), the plot of the mean particle size exhibited a parabolic shape, where 0.28 mM caffeic acid resulted in the smallest particle size of 89.4 nm. The mean particle size increased when the concentration of caffeic acid was either higher or lower than 0.28 mM. In particular, the mean particle size was exceptionally high (∼50 *μ*m) when the concentration of caffeic acid was 0.008 mM, and this result was not included in the curve fitting shown in (Fig. 4). The results presented in Fig. 4 demonstrate that 0.28 mM was the optimal concentration of caffeic acid to produce the smallest size of particles. In addition to the mean particle size, the zeta potential was also measured. The zeta potential was decreased from + 0.91 mV to −21.54 mV with increasing concentrations of caffeic acid, which may be the result of the addition of negative charges from caffeic acid (Table 1).

**Table 1:** Mean particle size and zeta potential values of AuNPs for various concentrations of caffeic acid (mM)

	0.008	0.04	0.08	0.12	0.16	0.2	0.24	0.28	0.4	0.48	0.56
Mean particle size (nm)	50238.4	895.5	619.6	423.1	153.4	109.6	95.4	89.4	22.6	209.1	624.2
Zeta potential (mV)	+ 0.91	−9.91	−11.35	−5.89	−14.17	−19.42	−16.5	−17.66	−15.68	−21.54	−16.8

### HR-TEM Images

HR-TEM is commonly employed to visualize nanoparticles and to obtain information regarding the size, shape, and dispersion state. The lowest concentration of caffeic acid (0.008 mM) was insufficient to reduce Au^3+^ to AuNPs and instead generated micro-sized particles with a mean particle size of >50 *μ*m (Additional file 1: Figure S2 (a)). As the concentration of caffeic acid increased from 0.04 to 0.24 mM, the mean particle size gradually decreased.

**Fig. 5:**
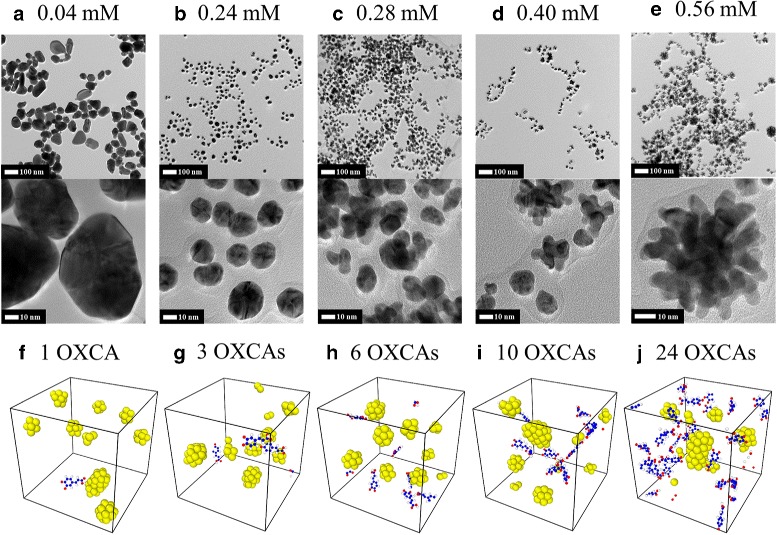
HR-TEM and MD simulation images of AuNPs. The final TEM image of caffeic acid is **a** 0.04, **b** 0.24, **c** 0.28, **d** 0.40, and **e** 0.56 mM and final MD atomic configuration is **f** 1, **g** 3, **h** 6, **i** 10, and **j** 24 OXCAs. The *scale bar* for each image represents 100 and 10 nm

Interestingly, the shape of the AuNPs became increasingly spherical as the concentration of caffeic acid increased up to 0.24 nm (Fig. 5a, b). The minimum particle size was observed at 0.28 mM, and a sea-urchin-like shape began to appear at this concentration (Fig. 5c). It is noteworthy that the mean particle size of the sea-urchin-like shape was larger when the caffeic acid concentration increased after 0.28 mM (Fig. 5d, e, Additional file 1: Figure S2 (e)). The same finding has been reported by Wang and co-workers when excessive ascorbic acid was added to HAuCl_4_ as a reducing agent [32]. The addition of excessive ascorbic acid generated sea-urchin-like Au particles with an average diameter of 600–800 nm.

### MD Results

**Fig. 6:**
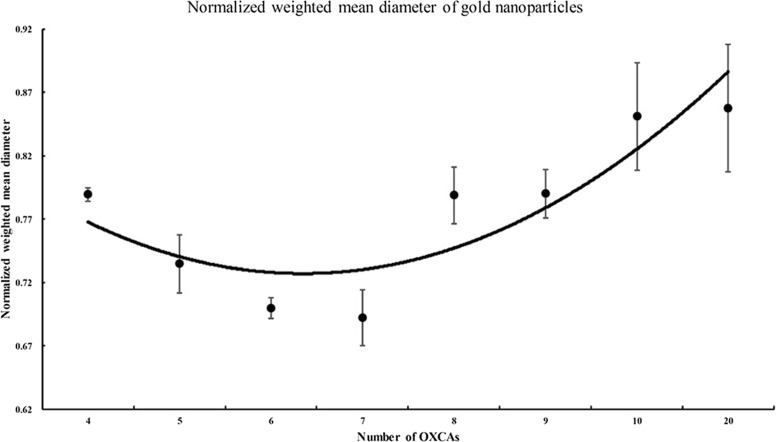
Normalized weighted mean diameter of gold nanoparticles with various numbers of reducing agents at 0.5 ns

The average of weighted mean diameters and its error analysis of aforementioned MD simulations for each case at the terminal time are listed in Table 2. The representative configurations of each case at terminal time of the MD simulations are shown in Fig. 5f–j. From the results, as shown in Fig. 6, we observe that there is a local minimum of average weighted mean diameters. The similar size of spherical shaped AuNPs were well-distributed over a wide area of simulation cell when seven reducing agents are used. When too many or too small number of reducing agents are used, relatively larger gold nanoparticles are aggregated. This tendency coincides with the results obtained from the experiment.

**Table 2:** Average of weighted mean diameters and error analysis

Number of OXCAs	Weighted diameter	STD	Standard error
1	5.40	0.53	0.24
2	5.41	0.36	0.16
3	5.37	0.69	0.31
4	5.65	0.08	0.04
5	5.26	0.36	0.16
6	5.01	0.13	0.06
7	4.95	0.35	0.16
8	5.64	0.36	0.16
9	5.65	0.31	0.14
10	6.09	0.68	0.30
20	6.13	0.80	0.36
24	7.15	0.23	0.10

### DFT Interpretation

**Fig. 7:**
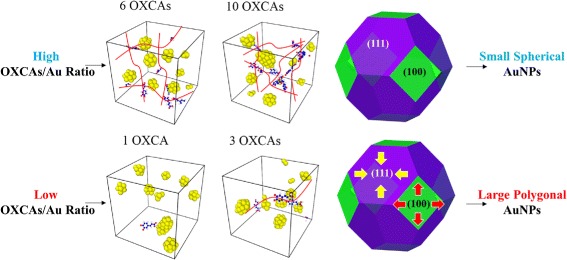
Size decrease and morphology change of AuNPs from low to high OXCA concentration

Using DFT calculations, we elucidate the mechanism of changes in the size and morphology of AuNPs as increasing the concentration of caffeic acid. At low concentration, the particle shapes are mostly controlled thermodynamically by surface free energies whose order is (111) < (100) < (110) (Table 3), due to the weak reducing effect. To minimize the surface energies in an Au nanoparticle, the low surface free energy facets such as {111} or {100} are dominantly exposed to form the polygonal particles as shown in Fig. 7. This is consistent with our observations from HR-TEM images (Fig. 5a, b) and MD simulations (Fig. 5f, g). At high concentration, the caffeic acid preferentially adsorbs on the surfaces of AuNPs. To understand in detail the effect of OXCA adsorption on the particle shape, we calculated the adsorption energies of OXCA and the OXCA-adsorbed surface free energies on Au (100), (110), and (111), respectively. The adsorption energy, *E*_*ads*_ (eV), on the different Au facets were calculated by

(5) $$\begin{array}{@{}rcl@{}} E_{ads} = E_{OXCA/surf} - E_{OXCA} - E_{surface}, \end{array} $$

**Table 3:** Calculated surface energies of Au(100), (111), and (110) surface with and without the adsorption of OXCA

Surface	(100)	(111)	(110)	*Δ* *φ* (100)–(111)	Diff (%)
*E*_*Surf*__*c* *l* *e* *a* *n*	0.325	0.284	0.567	0.041	
*E*_*Surf*__*O* *X* *C* *A*	0.310	0.276	0.549	0.034	17

where *E*_*OXCA*/*surf*_,*E*_*surface*_, and *E*_*OXCA*_ refer to the total energies of the surface with the adsorbed OXCA molecule, the optimized bare surface, and the isolated OXCA molecule in the gas phase, respectively. The surface free energy, *E*_*surf*_ (eV/atom), at the different Au facets were calculated by [33]

(6) $${} {\begin{aligned} E_{surf\_OXCA} &= \frac{1}{{N_{surf} }}\left[{\vphantom{\frac{1}{N_{surf}}}}\left({E_{substrate} - n \times E_{bulk}} \right)\right.\\ &\quad- E_{absorbate} - \left.\frac{1}{2}\left({E_{slab} - n \times E_{bulk}} \right)\right], \end{aligned}}  $$

where *E*_*substrate*_ is the total energy of the surface covered by the OXCA molecule, *E*_*slab*_ is the total energy of the fixed surface at their bulk atomic positions, *n* is the number of slab atoms, *E*_*bulk*_ is the chemical potential of Au fcc bulk phase, *N*_*surf*_ is the number of supercell atoms, *E*_*slab*_ is the total energy of clean surface [(111), (100) and (110)], and *E*_*adsorbate*_ is the total energy of isolated OXCA molecule or Au atom. In the case of clean surface energy calculations, *E*_*substrate*_ is changed to the total energy of the clean surface and *E*_*adsorbate*_ term is deleted as

(7) $$ \begin{aligned} E_{surf\_claen} &= \frac{1}{{N_{surf} }}\left[{\vphantom{\frac{1}{{N_{surf} }}}}\left({E_{substrate} - n \times E_{bulk}} \right) \right.\\ & \left. \quad- \frac{1}{2}\left({E_{slab} - n \times E_{bulk}} \right)\right], \end{aligned}  $$

**Fig. 8:**
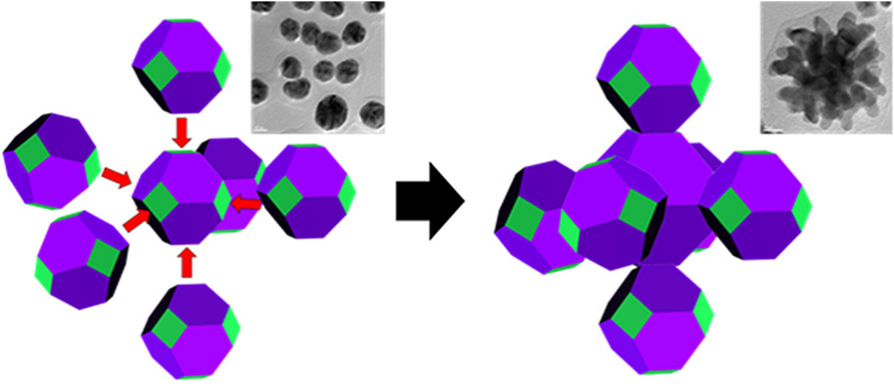
Schematic illustration of the particle growth mechanism in the sea-urchin-like shapes

The OXCA is more strongly adsorbed on the (110) surface of AuNP (−1.95 eV) than on (100) (−1.41 eV) and (111) (−0.94 eV), respectively (Additional file 1: Figure S4). The order of OXCA-adsorbed surface free energy is the same as that of clean surface free energy ((111) < (100) < (110)), but the difference in the surface free energies of two dominant surfaces, *Δϕ* (100)-(111), is decreased by 17 % (Table 3). The positive value of the difference indicates that the adsorbed OXCA reduces the surface energy of 100 facet, thereby suppressing the anisotropic growth toward [111] direction. In Fig. 8, the result from Wulff construction also describes that as the OXCA concentration increases, the morphology changes from the polygonal shape to the spherical shape with expanding the {100} facets.

In the previous sections, our experiments showed that once the concentration of caffeic acid increases, the size of AuNPs decreases until the concentration reaches to 0.28 mM. Recently, Yu et al. reported that branched polyethyleneimine (BPEI) acts as a reducing agent, capping agent, and stabilizer during the synthesis of AuNPs whose size can be controlled by a BPEI/HAuCl_4_ ratio [34]. In our case, the variation of particle size with increasing the concentration of caffeic acid can be interpreted in terms of OXCA’s multiple function similar to the BPEI. Basically, the caffeic acid reduces Au ions with inducing the aggregation of AuNPs. At the same time, however, the oxidation of caffeic acid also facilitates the adsorption of OXCA, resulting in the inhibition of the particle growth as schematically shown in Fig. 7. The competition between these two counteracting effects of caffeic acid determines the size of AuNPs. The concentration control of caffeic acid therefore plays an important role in the size of AuNPs.

To figure out the growth of AuNPs in the sea-urchin-like shape (Fig. 5e), we calculated the adsorption energies of an Au atom on the three different facets. The threefold follow sites are the most stable for forming the Au adatom with the adsorption energies of −3.04, −2.96, and −2.58 eV on (100), (110), and (111), respectively. The results show that (100) surface has the strongest affinity of an Au atom, and thus the particle growth is much preferred in the [100] direction to the other facets (Table 4). As mentioned above, the particle shape changes to a spherical shape due to the increase of {100} facets at the high concentration of OXCA. The well-distributed spherical AuNPs may preferentially bind each other by sharing the (100) crystal planes as schematically shown in Fig. 8. This anisotropic growth toward [100] therefore results in the formation of sea-urchin-like AuNPs.

**Table 4:** DFT-calculated binding energies of an Au single atom on Au (100), Au (110), and Au (111), respectively. Specific adsorption sites and configurations refer to Additional file 1: Figure S3

Surface	Top	Bridge	Hollow
100	−1.73	−2.36	−3.04
111	−1.84	–	−2.54(3H) −2.58(3F)
110	−1.64	−2.17(SB) −2.55(LB)	−2.96

## Conclusions

The concentration of caffeic acid plays an important role to control the size and morphology of AuNPs. Based on our results, we investigate the growth mechanism for the green synthesis of AuNPs. (1) At low concentration of caffeic acid, the shapes of AuNPs were thermodynamically controlled to polygonal shapes mostly exposed with {111} facets. (2) As the concentration of caffeic acid increases, the sizes of AuNPs were decreased due to the adsorption and stabilizing effect of OXCAs. The small size of spherical AuNP seeds starts to be generated. (3) The small size of seeds is preferentially bound to the {100} facet. These selective growth facilitates the rate of NP aggregation along the [100] direction, resulting in the formation of the sea-urchin-like AuNPs.

## Additional file

Additional file 1 Randomly packed initial configurations of Au particles at each concentration of OXCA. Yellow, red, white, and blue colors correspond to gold, oxygen, hydrogen, and carbon atoms, respectively (**Figure S1**). HR-TEM images of AuNPs. The final concentration of caffeic acid is (a) 0.008 mM, (b) 0.08 mM, (c) 0.12 mM, (d) 0.16 mM, (e) 0.20 mM and (f) 0.48 mM and the scale bar for each image represents 100 nm and 10 nm (**Figure S2**). Considered adsorption sites and the most stable adsorption configurations of Au adatom on Au(111), (110) and (100) surfaces, respectively (**Figure S3**). The most stable adsorption configurations and energies of OXCA on Au(111), (110), and (100) surfaces, respectively (**Figure S4**). (PDF 796 kb)
