# Supplementary material for: Concentration Effect of Reducing Agents on Green Synthesis of Gold Nanoparticles: Size, Morphology, and Growth Mechanism
Source: Nanoscale Res Lett. 2016 Apr 27;11:230. doi: 10.1186/s11671-016-1393-x (PMC4848276; doi:10.1186/s11671-016-1393-x)
Supplement: Additional file 1 — Randomly packed initial configurations of Au particles at each concentration of OXCA. Yellow, red, white, and blue colors correspond to gold, oxygen, hydrogen, and carbon atoms, respectively (Figure S1). HR-TEM images of AuNPs. The final concentration of caffeic acid is (a) 0.008 mM, (b) 0.08 mM, (c) 0.12 mM, (d) 0.16 mM, (e) 0.20 mM and (f) 0.48 mM and the scale bar for each image represents 100 nm and 10 nm (Figure S2). Considered adsorption sites and the most stable adsorption configurations of Au adatom on Au(111), (110) and (100) surfaces, respectively (Figure S3). The most stable adsorption configurations and energies of OXCA on Au(111), (110), and (100) surfaces, respectively (Figure S4). (PDF 796 kb) [file 11671_2016_1393_MOESM1_ESM.pdf]

Concentration Effect of Reducing Agents on Green Synthesis of Gold Nanoparticles: Size, Morphology, and Growth Mechanism

Hyun-Seok Kim[1], *Yu Seon Seo*[2], *Kyeounghak Kim*[3], *Jeong Woo Han*[3], *Youmie Park*[2], *and Seonho Cho*[1]

[1]National Creative Research Initiatives (NCRI) Center for Isogeometric Optimal Design, Seoul National University, 1 Gwanak-ro, Gwanak-gu, Seoul, 151-744, Republic of Korea.

[2]College of Pharmacy, Inje University, 197 Inje-ro, Gimhae, Gyeongnam, 621-749, Republic of Korea.

[3]Department of Chemical Engineering, University of Seoul, Seoul, 130-743, Republic of Korea.

* To whom correspondence should be addressed: E-mail: secho@snu.ac.kr (S.Cho)

In order to figure out the size and morphology of AuNPs at different concentration of reducing agent (caffeic acid), we performed both experimental and theoretical approaches. Our MD simulation results showed that the particle size and morphology were controlled by the concentration of caffeic acid. The growth and morphology of AuNPs were thermodynamically explained by the adsorptions of Au atoms and OXCA, respectively. For more information, we present more data; initial configuration of Au particles at each concentration of OXCA for MD simulations (Figure S1), HR-TEM images of AuNPs at various concentrations of OXCA (Figure S2), and considered adsorption sites and configurations for DFT calculations (Figure S3).

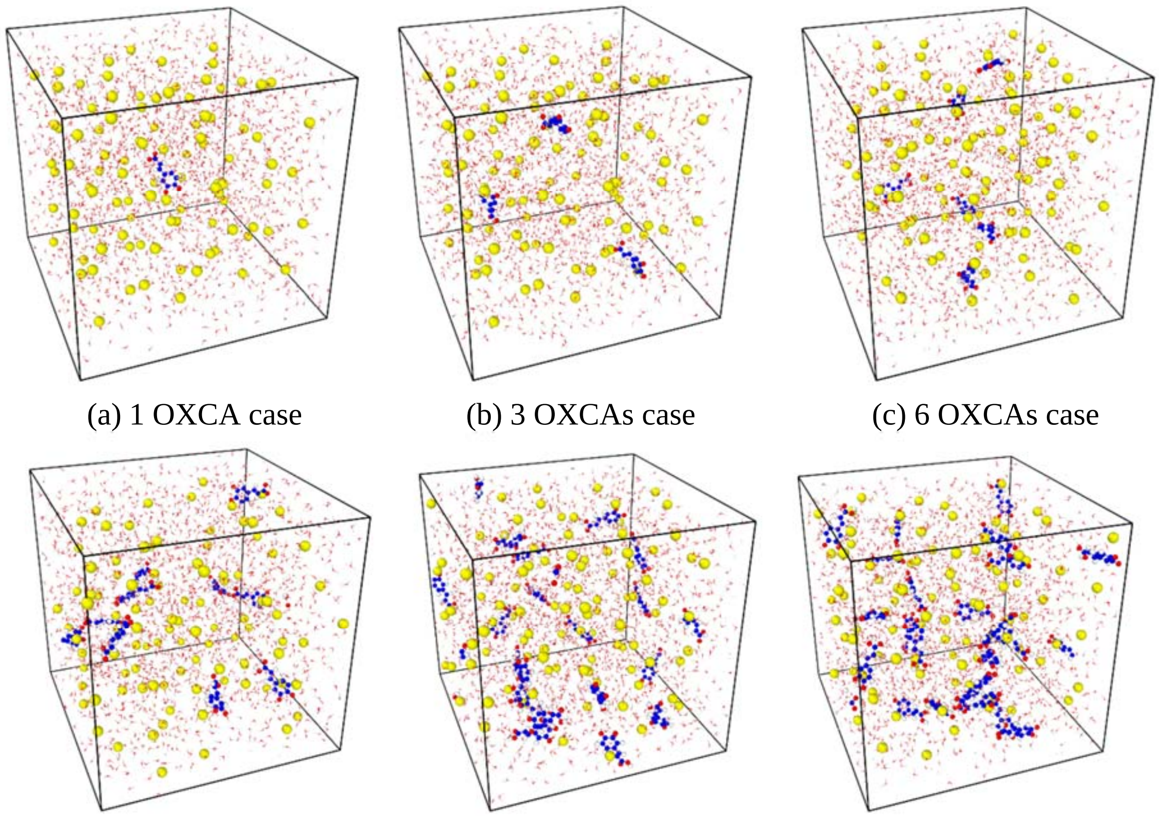

(a) 1 OXCA case (b) 3 OXCAs case (c) 6 OXCAs case

(d) 10 OXCAs case (e) 20 OXCAs case (f) 24 OXCAs case

Figure S1. Randomly packed initial configurations of Au particles at each concentration of OXCA. Yellow, red, white, and blue colors correspond to gold, oxygen, hydrogen, and carbon atoms, respectively.

To minimize the dependence of initial configurations on the MD simulations, the simulations are performed many times and the caffeic acid molecules and Au monomers are evenly distributed into the cube unit cells (Figure S1).

We examined the size and morphology of AuNPs as varying the concentration of caffeic acid from 0.008 to 0.48 mM. In addition to the figures included in this manuscript, HR-TEM images of AuNPs at the other concentrations of caffeic acid also support the suggested mechanism (Figure S2); the particle sizes were obviously decreased form 0.08 mM to 0.20 mM while the AuNP's aggregation was occurred at 0.48 mM.

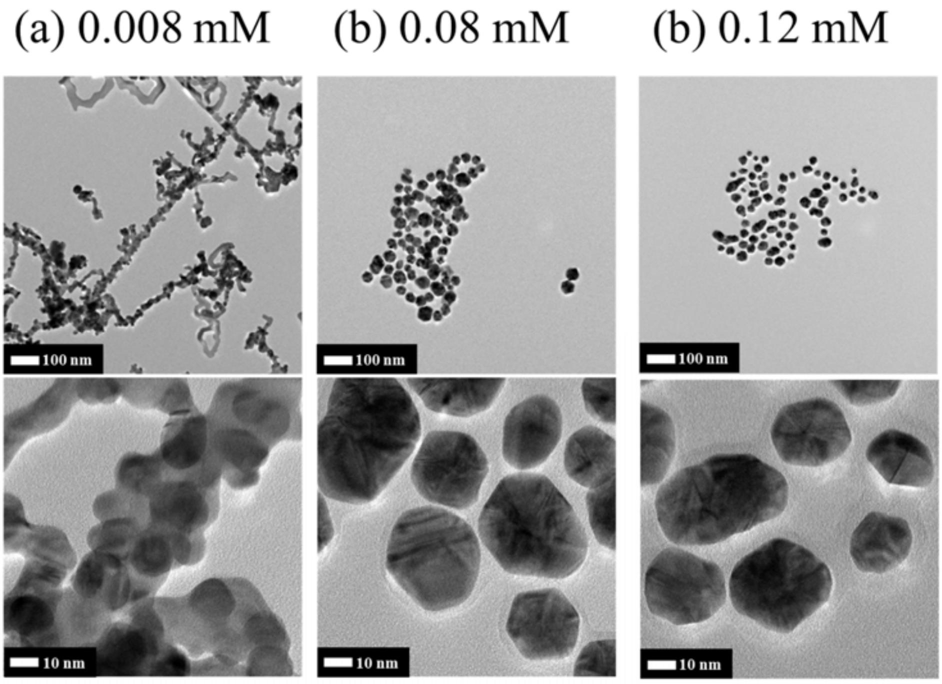

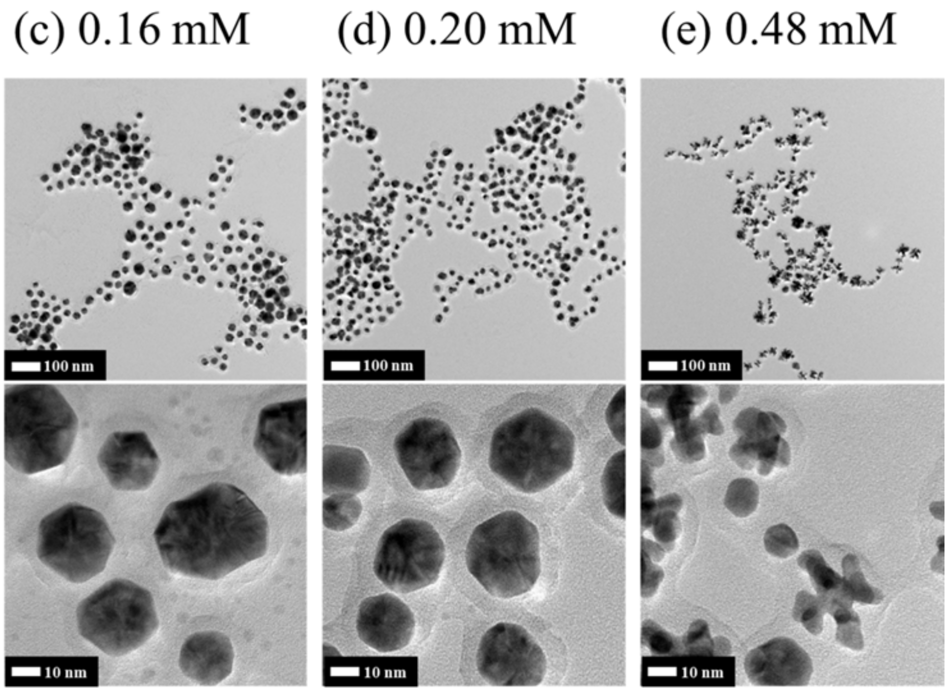

Figure S2. HR-TEM images of AuNPs. The final concentration of caffeic acid is (a) 0.008 mM, (b) 0.08 mM, (c) 0.12 mM, (d) 0.16 mM, (e) 0.20 mM and (f) 0.48 mM and the scale bar for each image represents 100 nm and 10 nm.

We considered all possible adsorption sites of Au adatom. The adsorption of Au adatom strongly favors the hollow sites on all Au facets (Figure S3).

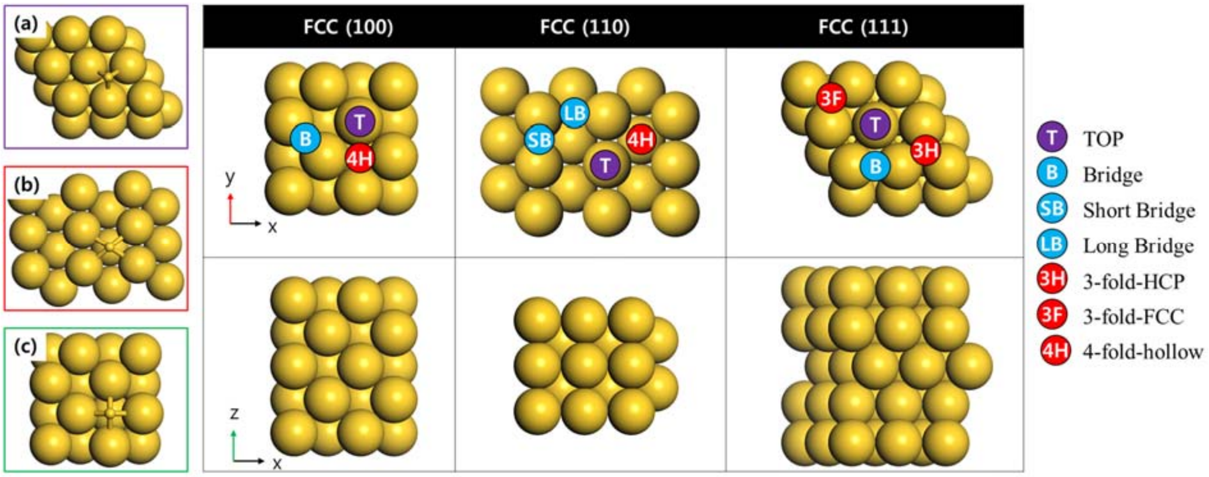

Figure S3. Considered adsorption sites and the most stable adsorption configurations of Au adatom on Au(111), (110) and (100) surfaces, respectively.

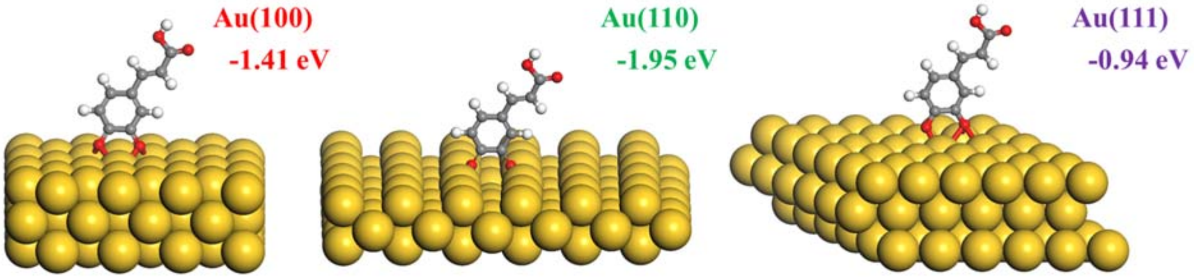

Figure S4. The most stable adsorption configurations and energies of OXCA on Au(111), (110), and (100) surfaces, respectively.

The MD results showed that OXCAs were adsorbed on the AuNPs mostly via the catechol functional group (Figure 5). The adsorption of OXCA via the other functional groups such as carboxyl group was also observed on the Au surfaces from the DFT calculations. However, the adsorption of OXCA via the catechol group is still more preferred than the others (Figure S4). In addition, our results indicated that Au (110) is mostly affected by the OXCA adsorption.
